# Social Welfare

**Published:** 1920-07-17

**Authors:** 


### Social Welfare.

### Programmes at Summer Schools.

We have received notice of two useful summer school programmes. The first is the Summer School of Eugenics and Civics to be held at Herne Bay College, Kent, from July 31 to August 18, under the auspices of the National Council for Combating Venereal Diseases. The courses include teaching of biology in schools and training-colleges; social psychology; heredity in relation to eugenics, etc. The Council also provides other facilities for instructing teachers and social workers on subjects of sex hygiene and venereal disease. It is convinced that if the true facts could be disseminated among the working teachers of the country, a strong opinion would be generated that instruction in these urgent matters should not be left to chance, but should form an integral part of the instruction given in the training-colleges. Although the new syllabus for hygiene teaching in these colleges has a wide range, instruction in sex hygiene and the dangers of venereal disease is still optional, and, leaving aside the vexed question of direct tuition in the schools, the Council considers that training-college students ought, at any rate, to be instructed.

During the same fortnight the Civic Education League will hold its summer school at High Wycombe, Bucks. The range of subjects has interest for all connected with social, educational, and health work, and there is a distinguished list of lecturers. Included in the programme will be short courses on sociology, social philosophy, social psychology, principles and practice of sex education, problems of the industrial system, and methods of teaching civics. The National League for Health, Maternity, and Child Welfare and the National Council of Social Service are co-operating. The Secretary's office is at 65 Belgrave Road, S.W. 1.

